# Supramolecular Conductive Hydrogels With Homogeneous Ionic and Electronic Transport

**DOI:** 10.1002/adma.202415687

**Published:** 2025-04-28

**Authors:** Stephen J.K. O'Neill, Minoru Ashizawa, Alan M. McLean, Ruben Ruiz‐Mateos Serrano, Tokihiko Shimura, Masakazu Agetsuma, Motosuke Tsutsumi, Tomomi Nemoto, Christopher D. J. Parmenter, Jade A. McCune, George G. Malliaras, Naoji Matsuhisa, Oren A. Scherman

**Affiliations:** ^1^ Melville Laboratory for Polymer Synthesis, Yusuf Hamied Department of Chemistry University of Cambridge Lensfield Road Cambridge CB2 1EW UK; ^2^ Department of Materials Science and Engineering Tokyo Institute of Technology 2‐12‐1 Ookayama, Meguro‐ku Tokyo 152‐8552 Japan; ^3^ Electrical Engineering Division, Department of Engineering University of Cambridge 9 JJ Thomson Ave Cambridge CB3 0FA UK; ^4^ Research Center for Advanced Science and Technology The University of Tokyo 4‐6‐1 Komaba, Meguro‐ku Tokyo 153‐8505 Japan; ^5^ Electronics and Electrical Engineering, Faculty of Science and Engineering Keio University 3‐14‐1 Hiyoshi, Kohoku‐ku, Yokohama Kanagawa 223‐8522 Japan; ^6^ Institute of Industrial Science The University of Tokyo 4‐6‐1 Komaba, Meguro‐ku Tokyo 153‐8505 Japan; ^7^ Division of Homeostatic Development National Institute for Physiological Sciences 38 Nishigohnaka Myodaiji‐cho, Okazaki Aichi 444‐8585 Japan; ^8^ Quantum Regenerative and Biomedical Engineering Team, Institute for Quantum Life Science National Institutes for Quantum Science and Technology (QST) Anagawa 4‐9‐1, Chiba Inage‐ku Chiba 263‐8555 Japan; ^9^ Biophotonics Research Group, Exploratory Research Center on Life and Living Systems National Institutes of Natural Sciences Okazaki Aichi 444‐8787 Japan; ^10^ Research Division of Biophotonics, National Institute for Physiological Sciences National Institutes of Natural Sciences Okazaki Aichi 444‐8787 Japan; ^11^ Nottingham Nanoscale and Microscale Research Centre University of Nottingham University Park Nottingham NG7 2RD UK

**Keywords:** bioelectronics, conducting polymers, host‐guest chemistry, reusable devices, supramolecular networks

## Abstract

Mechanically resilient hydrogels with ion‐electron mixed transport properties effectively bridge biology with electronics. An ideal bioelectronic interface can be realized through introducing electronically conductive polymers into supramolecular hydrogels. However, inhomogeneous morphologies of conducting polymers, such as poly(3,4‐ethylenedioxythiophene):poly(styrene sulfonate) (PEDOT:PSS), have limited mechanical properties and ion‐electron interactions. Here, supramolecular conductive hydrogels that possess homogeneous ionic and electronic transport are achieved. The materials demonstrate high toughness (620 kJ m^−3^), stretchability (>1000%), softness (10.5 kPa), and conductivity (5.8 S cm^−1^), which surpasses commonly used inhomogeneous PEDOT:PSS‐based hydrogels. The homogeneous network leads to higher charge injection capacitance and lower skin impedance compared to commercial electrodes or commonly used inhomogeneous PEDOT:PSS conducting networks. This significant advance arises from the homogeneous incorporation of the hydrophilic self‐doped conducting polymer S‐PEDOT, which has polymerized within a supramolecular polymer network template mediated by high‐binding affinity host‐guest crosslinks. Furthermore, the compatibility of S‐PEDOT with hydrophilic secondary networks enables the realization of fully dryable and reswellable electronic devices, facilitating reusability and improving their ease of handling. It is anticipated that achieving such material architectures will offer a promising new direction in future synthesis and implementation of conductive hydrogels in the field of bioelectronics.

## Graphical abstract

Poly(3,4‐ethylenedioxythiophene) polystyrene sulfonate (PEDOT:PSS) based conductive hydrogels have received great attention in bioelectronics on account of their tissue‐like mechanical properties. However, inhomogeneous morphologies of the conducting PEDOT phase limits their electrical and mechanical properties. Here, supramolecular hydrogels with self‐doped PEDOT (S‐PEDOT) homogeneously distributed are reported, which simultaneously exhibit high toughness (620 kJ m^−3^), softness (10.5 kPa) and conductivity (5.8 S cm^−1^).

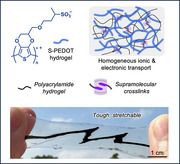

## 1 Introduction

Bioelectronic interfaces mechanically and electrically bridge biology with electronics, required for medical implants, epidermal electronics, and neuroprosthetics.^[^ ^1^, ^2^, ^3^, ^4^ ^]^ Durable mechanical properties are essential to ensure conformal contact with biological organs for long‐term integration.^[^ ^5^, ^6^, ^7^ ^]^ Additionally, the materials should possess mixed transportation capability of ions and electrons, which are the information carriers for biology and electronics, respectively.^[^ ^8^, ^9^, ^10^ ^]^ Hydrogels are a highly suitable class of materials on account of their tissue‐mimetic attributes (high water contents, viscoelastic, soft).^[^ ^11^, ^12^, ^13^, ^14^ ^]^ In particular, supramolecular hydrogels based on dynamic cucurbit[n]uril (CB[n]) host‐guest chemistry can offer exceptional mechanical properties, including tissue‐like self‐healing and high toughness, realized through non‐covalent crosslinking between polymer chains.^[^ ^15^, ^16^, ^17^ ^]^

Electronic conducting networks have been introduced to ionically conductive hydrogels by employing conductive polymers such as PEDOT:PSS.^[^ ^18^, ^19^, ^20^, ^21^, ^22^ ^]^ Recently, high electronic conductivities were achieved through micro‐phase separation of PEDOT in less hydrophilic mechanical networks (such as polyurethane).^[^ ^23^ ^]^ For improved ion‐electron mixed transport and mechanical properties, homogeneous morphologies of conducting polymers are ideal rather than heterogeneous structures.^[^ ^24^ ^]^ A recent study utilized polyacrylic acid (PAA) based hydrogels as pre‐templates for achieving homogeneous morphologies of conducting polymers.^[^ ^25^ ^]^ Increased interfacial area between the electrolyte and conjugated polymer gives larger magnitudes of ionic–electronic coupling and volumetric capacitance.^[^ ^26^ ^]^ A further study mitigated the effects of inhomogeneity by additionally introducing polyaniline (PANI), to interconnect the aggregated phases of PEDOT.^[^ ^27^ ^]^

Achieving homogeneous ion‐electron mixed transport in mechanically durable and functional hydrogel templates, especially those based on hydrophilic polymers, has been challenging. This is on account of the highly hydrophobic PEDOT, which is not readily compatible with hydrogels. Recently, high conductivities of over 1,000 S cm^−1^ were reported in hydrophilic self‐doped PEDOT (S‐PEDOT) dry thin films, which displayed high dispersibility in water.^[^ ^28^ ^]^ Furthermore, we recently reported a poly(dimethylacrylamide) hydrogel containing a newly designed CB[8] supramolecular cross‐link (CB[8]·(BPyVI)_2_) with ultra‐high binding affinities, which maintained exceptional mechanical properties after in situ synthesis of a conducting polymer (PEDOT:PSS).^[^ ^29^ ^]^ We hypothesized that these two systems would allow for the homogeneous formation of conductive polymer networks within supramolecular hydrogels.

**Figure 1:**
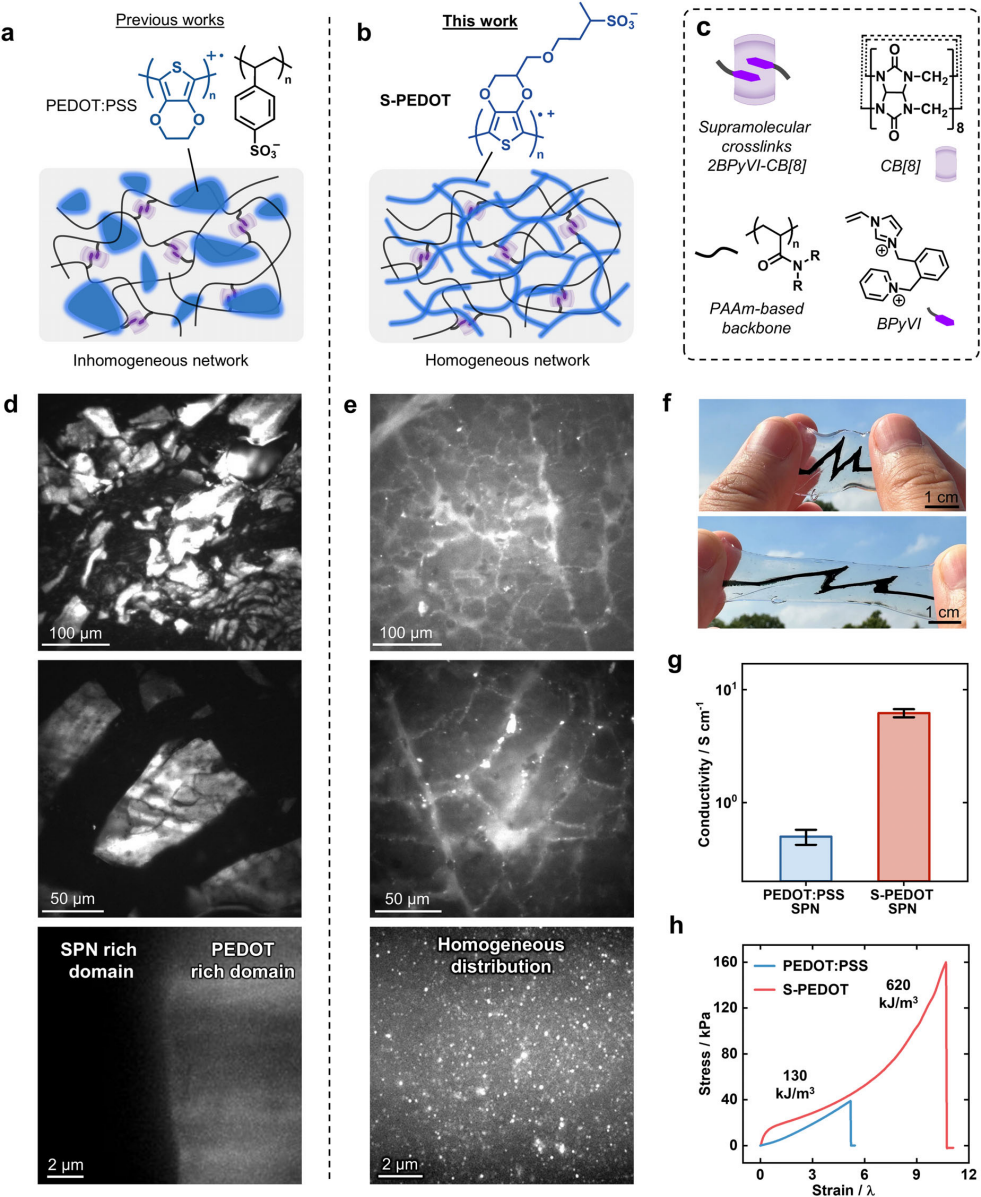
Highly Homogeneous Conducting Networks using S‐PEDOT. a) Schematic illustration of previous works using PEDOT:PSS conducting polymers, showing inhomogeneity and PEDOT aggregated domains. b) Schematic illustration of this work using S‐PEDOT, showing improved homogeneity and network formation of the conducting polymer. c) Molecular design of the SPN, incorporating CB[8] supramolecular ternary complexes as dynamic cross‐links. d) Fluorescence microscopy images of the PEDOT:PSS SPN, in which there are visible fluorescent PEDOT domains inhomegenuously dispersed. e) Fluorescence microscopy images of the S‐PEDOT SPN, showing improved homogeneity of S‐PEDOT without visible domain separation. f) Images of the S‐PEDOT SPN on a SPN substrate under strain. g) Conductivity comparison of the S‐PEDOT and PEDOT:PSS SPNs. Statistical significance was determined by one‐way ANOVA and Tukey's multiple comparison test (*P* < 0.0001). h) Tensile stress–strain curves of the S‐PEDOT and PEDOT:PSS SPNs.

Here, we demonstrate homogeneous conducting polymer networks in supramolecular hydrogels to simultaneously achieve high toughness (620 kJ m^−3^), stretchability (>1000%), softness (10.5 kPa), and electronic conductivity (5.8 S cm^−1^). The homogeneous S‐PEDOT networks further show lower skin impedance and higher charge injection capacitance (1.41 mC cm^−2^) compared to commercial electrodes or analogous PEDOT:PSS hydrogels with heterogeneous structures. The improved homogeneity can be attributed to the charged sulfonic‐acid side groups in S‐PEDOT, increasing the hydrophilicity compared to PEDOT, **Figure** **1**a,b. Furthermore, the improved compatibility of S‐PEDOT with hydrophilic mechanical networks enables the fabrication of fully dryable and re‐swellable hydrogel devices, improving their ease of handling, implementation and reusability.

## 2 Design and Characteristics of the S‐PEDOT SPN

The homogeneous conductive networks were prepared via redox polymerization of self‐doped EDOT monomer (S‐EDOT) within a supramolecular polymer network (SPN) template, more details are available in Figures S1–S5 (Supporting Information). Increased hydrophilicity of S‐PEDOT in comparison to PEDOT enables integration with a highly stretchable and tough polyacrylamide mechanical network employing supramolecular cross‐links, Figure 1c. The high solubility of S‐EDOT monomer in water facilitates polymerization within the hydrogels native aqueous environment without the use of organic solvents. In contrast, EDOT monomer requires ethanol for effective in situ synthesis to PEDOT:PSS on account of it's hydrophobicity, limiting the mechanical network to less hydrophilic polymers that show inferior mechanical properties to hydrophilic polymers such as polyacrylamide.^[^ ^28^, ^30^, ^31^ ^]^

A highly homogeneous network of S‐PEDOT was observed across the sample through the use of fluorescence microscopy, Figure 1e, whereby fluorescence arising from the conjugated polymer could be detected.^[^ ^32^ ^]^ In low magnification the S‐PEDOT appears to form a network structure. At high magnification (100x) fluorescence from the S‐PEDOT can be observed throughout the sample (the background), although the appearance of homogeneity was affected by typical single‐molecule imaging artefacts of photobleaching and inhomogeneous broadening due to the microscope and ultra‐zoom used (not present in 10x and 20x samples).^[^ ^33^ ^]^ In contrast, an analogous supramolecular network with PEDOT:PSS in poly(dimethylacrylamide) showed inhomogeneous aggregation and domain separation of PEDOT, Figures 1d. PEDOT‐rich domains of approximately 50 microns could be observed with limited interconnectivity. The S‐PEDOT and PEDOT:PSS network morphologies were further investigated at higher resolutions using cryo‐focused ion beam scanning electron microscopy (cryo‐FIB SEM), however, the relative distributions of PEDOT for each sample could not be observed in the dry, porous state (Figure S6, Supporting Information).

The observation of electronic network formation in SPNs is consistent with the electrical conductivity. The S‐PEDOT SPN showed over an order of magnitude increase in conductivity compared to the PEDOT:PSS SPN, from 0.52 S to 5.8 S cm^−1^, Figure 1g. The increased homogeneity and network formation gave rise to interconnected electron conduction pathways within the S‐PEDOT, as opposed to restricted mobility between PEDOT domains.^[^ ^34^ ^]^ The conductivity of 5.8 S cm^−1^ is reasonably scaled from that of the dry film of S‐PEDOT on account of the homogeneity in the matrix. The conductivity of dry S‐PEDOT was measured to be roughly 150 S cm^−1^, and the weight fraction of S‐PEDOT in the SPN is estimated to be 7.3 wt.%. Meanwhile, the weight fraction of PEDOT:PSS within the PEDOT:PSS SPN is estimated to be 8.9 wt.%. The weight percent of the conducting polymer was approximated by weighing the dry sample before and after in situ polymerization of the conducting polymer. The value calculated is just an approximation, as there may be some proportion of weight deriving from residual counterions within the sample.

Compared to the S‐PEDOT SPN, a significantly higher conductivity of over 200 S cm^−1^ using PEDOT:PSS at only 1 wt.% has been previously reported,^[^ ^25^ ^]^ however, at this low wt.% it does not scale appropriately with percolation theory nor does it fall in‐line with previously reported values in the field. The same material composition (PAA/PEDOT:PSS) has been separately reported to have a conductivity of 0.5 S cm^−1^, while pure PEDOT:PSS hydrogels have a reported conductivity of 40 cm^−1^.^[^ ^18^, ^21^ ^]^ Nevertheless, achieving the highest reported conductivity is not the main aim of our work, and the S‐PEDOT SPN conductivity is suitable for bioelectronic applications. Moreover, conductive hydrogels are typically used at a thickness greater than 100 µm, for which the sheet resistance of the S‐PEDOT SPN is at least 17 Ω, more than two orders of magnitude lower than the impedance of biological tissue.^[^ ^35^, ^36^ ^]^ For example, the S‐PEDOT SPN conductivity is sufficient for large, cutaneous electrodes with short conductive tracks to a bioelectronic device. Meanwhile for microfabricated electrodes implanted deep into the body, metal tracks would be needed to achieve an overall lower impedance.

The S‐PEDOT SPN also showed a significant improvement in mechanical properties compared to an analogous PEDOT:PSS SPN. The toughness increased from 130 kJ to 620 kJ m^−3^, and the stretchability increased from 5.2 to 10.7 elongation ratio, while the Young's modulus remained low at 10.5 kPa, similar to that of cardiac tissue (8 kPa),^[^ ^37^, ^38^ ^]^ Figure 1f. The improved mechanical properties are likely due to an interpenetrating network toughening effect,^[^ ^14^ ^]^ which would not occur in the case of aggregated PEDOT domains with poor interpenetration of polymers.

**Figure 2:**
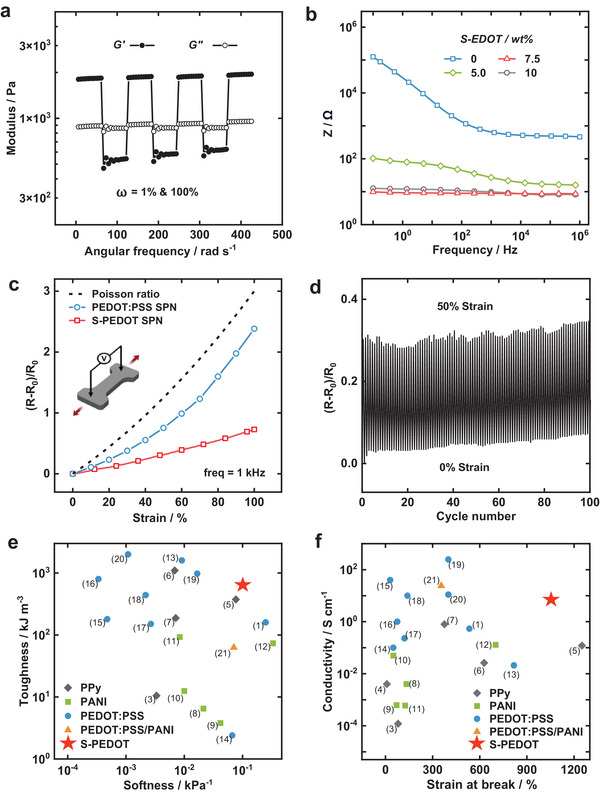
Mechanical & Electrical Characterisation of the S‐PEDOT SPN. a) Rheological continuous step–strain measurements at 20 °C of the S‐PEDOT SPN. b) Bode impedance plot of the S‐PEDOT SPN networks with varying S‐EDOT monomer concentrations. c) Impedance of the S‐PEDOT SPN and PEDOT:PSS SPN at 1 kHz during tensile deformation. d) Resistance change versus tensile cycle of the S‐PEDOT SPN. e) Comparison plot of softness and toughness of the S‐PEDOT SPN compared to previous reports of conducting polymer hydrogels. f) Comparison plot of stretchability and conductivity of the S‐PEDOT SPN compared to previous reports of conducting polymer hydrogels. The numbers on (e & f) are in relation to Table S1 (Supporting Information).

Oscillatory rheology was employed to investigate the viscoelastic properties of the network, in which it was observed that the supramolecular cross‐links were not disrupted by the S‐PEDOT. Subjecting the S‐PEDOT SPN to multiple step‐strain cycles (1% & 100% strain at 1 rad s^−1^) led to a reversible sol–gel transition, indicative of energy dissipation and self‐recovery processes due to reversible association and dissociation of cross‐links, **Figure** **2**a.^[^ ^39^, ^40^, ^41^, ^42^ ^]^ A slight increase in storage and loss moduli was observed (6% and 10%, respectively) during cycling, which may be attributed to drying of the SPN during the experiment. A frequency and amplitude sweep were also performed, displaying a frequency‐dependent modulus, tissue‐like viscoelasticity and high amplitude strain tolerance, characteristic of supramolecular cross‐linking, Figure S7 (Supporting Information).^[^ ^15^, ^43^, ^44^ ^]^

The high electronic conductivity was confirmed by frequency‐dependent electrochemical impedance spectroscopy of S‐PEDOT SPNs with various concentrations of S‐EDOT monomer during in situ polymerization, Figure 2b. The direct current (DC) measurement can lead to an overestimation of conductivity because of capacitive charging or water splitting at the interface between electrodes and the hydrogel. In the absence of S‐PEDOT, the supramolecular mechanical network shows a frequency‐dependent capacitive response with higher impedance at lower frequencies (<10^3^ Hz), on account of limited electron mobility.

As the S‐EDOT wt.% is increased, the impedance decreases and becomes less frequency dependent, indicative of electron mobility and high conductance due to the formation of an electronic network. The 7.5 wt.% sample showed the highest conductivity, with no frequency dependence in the impedance ranging from 0.1 Hz to 1 MHz, and was therefore chosen for subsequent experimental characterization and application investigation.

The S‐PEDOT electrical properties are also considerably more stable compared to the PEDOT:PSS SPN under tensile strain, Figure 2c. Under 100% strain, the increase in impedance of the S‐PEDOT SPN is less than 80%, while the PEDOT:PSS SPN impedance increases by 230%. The improved stability is likely due to continuous electron conduction pathways of S‐PEDOT, while the PEDOT:PSS SPN suffers from disconnections of the aggregated PEDOT phases with strain application. Additionally, under cyclical deformation, the impedance change of the S‐PEDOT SPN remained stable, with less than 10% deviation over 100 cycles to elongation strains of 50%, which is slightly higher than the stretchability of human skin, Figure 2d.^[^ ^45^ ^]^

The conductivity and mechanical properties of the S‐PEDOT SPN have been comprehensively compared to previous reports of conducting polymer‐based hydrogels in Figures 2e,f and Table S1 (Supporting Information). In general, hydrogels exhibit a toughness‐softness trade‐off, where tougher hydrogels are typically stiffer. Impressively, the S‐PEDOT SPN achieves a level of both high toughness and high softness simultaneously, which can be attributed to energy dissipation effects of the high‐binding affinity host‐guest cross‐links.^[^ ^15^ ^]^ The softness is defined as the inverse of the Young's modulus. In addition, hydrogels with higher electrical conductivity generally display a lower stretchability, demonstrating an intrinsic trade‐off between mechanical properties and conductivity. The S‐PEDOT SPN overcomes this trade‐off, achieving a high stretchability (>10 times extension), while maintaining a high conductivity (>5 S cm^−1^). Such a combination of properties is owing to the fact that there are no aggregated hard domains of conjugated polymer, and S‐PEDOT is homogenously dispersed in a tough supramolecular hydrogel.^[^ ^18^ ^]^ Forming homogeneous conducting networks without limitations on the specific hydrogel template further unlocks the potential for new properties, achieved through tailoring the molecular structures of the hydrogel.

## 3 Dryable and Reswellable Networks

The improved compatibility of S‐PEDOT with hydrophilic hydrogel templates enables dryable and rapidly reswellable electronically conductive polymer hydrogels. Reswellable hydrogels have been utilized for applications such as drug delivery, biosensing, actuators, and cosmetic applications.^[^ ^46^, ^47^, ^48^ ^]^

Removing water makes hydrogels easier to handle and transport, as it reduces their tendency for shape‐morphing and unwanted adhesion to surfaces. Furthermore, the ability the reswell the networks allows for reusability of the devices after their application and dehydration. The dry S‐PEDOT SPNs are also 83% lighter than in their hydrated state, reducing transportation costs, and environmental implications.

**Figure 3:**
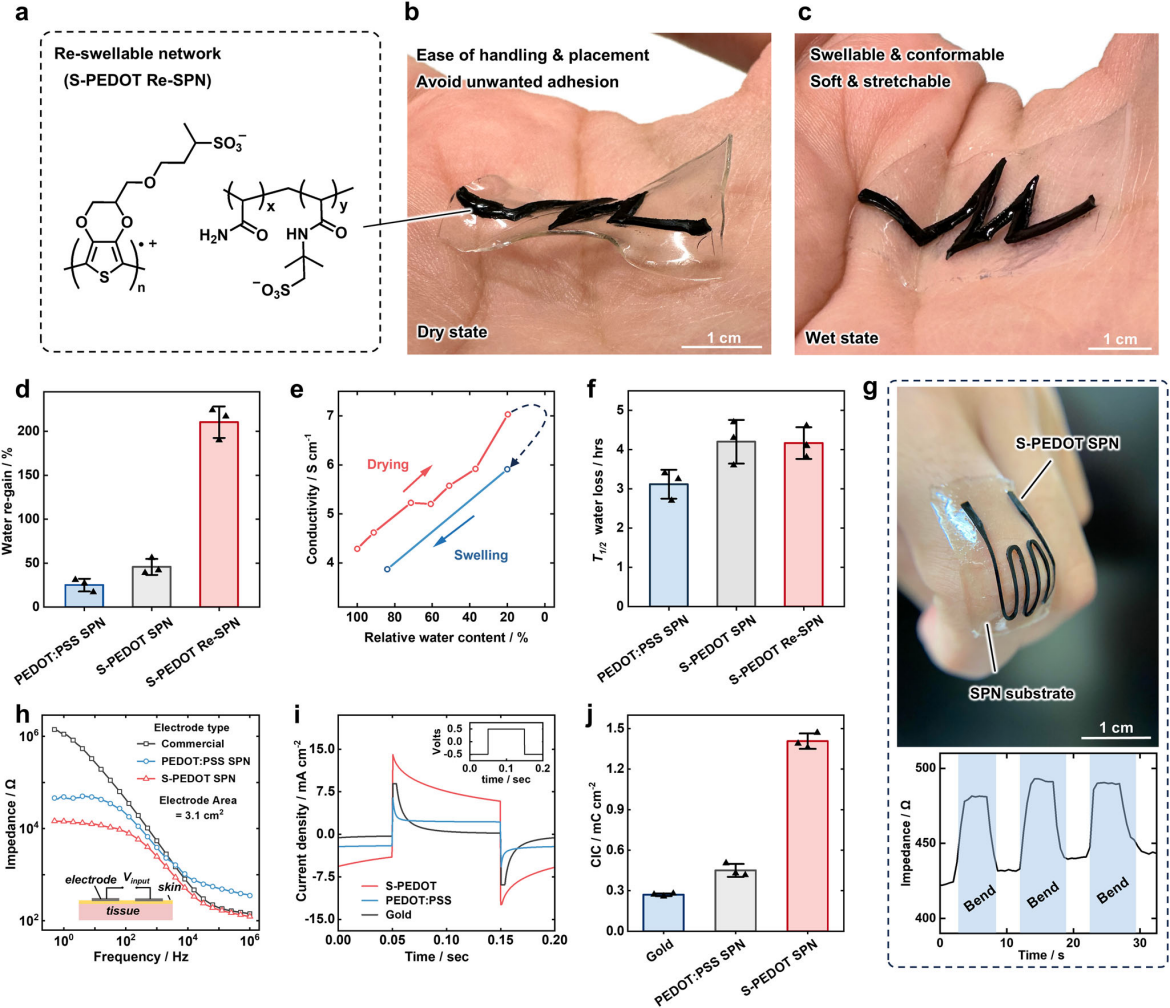
S‐PEDOT SPN Bioelectronic Applicability. a) Molecular design of the S‐PEDOT Re‐SPN, incorporating sulfonic acid to the SPN. b c) Images of the laser patterned S‐PEDOT Re‐SPN and Re‐SPN substrate undergoing a drying and re‐swelling process. d) Swelling degree of the S‐PEDOT and PEDOT:PSS SPN. Statistical significance between PEDOT:PSS SPN and S‐PEDOT Re‐SPN was determined by one‐way ANOVA and Tukey's multiple comparison test (*P* < 0.0001). e) Conductivity measurements during drying and re‐swelling of the S‐PEDOT SPN. f, Drying half‐time of the S‐PEDOT and PEDOT:PSS SPN. Statistical significance between PEDOT:PSS SPN and S‐PEDOT Re‐SPN was determined by one‐way ANOVA and Tukey's multiple comparison test (*P* < 0.03) g) Image of the laser‐cut strain sensor adhered on the fingertip of a human hand (above) and real‐time resistance of the strain sensor with strain (below). h) Skin impedance Bode plot of the S‐PEDOT SPN, PEDOT:PSS SPN, and commercial Kendall electrode. i) Biphasic input pulses (inset) and the corresponding current density versus time plots for the S‐PEDOT SPN, PEDOT:PSS SPN and gold electrode. j) Charge injection capacitance for the S‐PEDOT SPN, PEDOT:PSS SPN and gold electrode. Statistical significance between PEDOT:PSS SPN and S‐PEDOT Re‐SPN was determined by one‐way ANOVA and Tukey's multiple comparison test (*P* < 0.0001).

Incorporating charged polymer backbones with dynamic cross‐links can endow rapid reswelling in hydrogels on account of the high polymer mobility and high polarity.^[^ ^49^, ^50^ ^]^ To achieve rapid reswellability, we incorporated the charged monomer 2‐Acrylamido‐2‐methylpropane sulfonic acid (AMPS) to the supramolecular polymer backbone, forming a copolymer with acrylamide at a ratio of 1:1, **Figure** **3**a. S‐PEDOT can be incorporated as previously with in situ polymerization, forming S‐PEDOT reswellablable supramolecular networks (S‐PEDOT Re‐SPN). The S‐PEDOT Re‐SPNs show a similarly high conductivity of 4.3 S cm^−1^. Further, the S‐PEDOT Re‐SPNs demonstrate a low tissue‐like Youngs modulus of 7.9 kPa, and significantly higher stretchability (910%) and toughness (305 kPa) compared to the PEDOT:PSS SPN, Figure S8 (Supporting Information). Conversely, PEDOT:PSS networks are limited to less hydrophilic mechanical polymers on account of the hydrophobicity of PEDOT and the low solubility of EDOT in aqueous solutions. Following drying of the S‐PEDOT Re‐SPN, immersion in water led to rapid re‐swelling to its original weight within 2 min, demonstrating reswellable conductive polymer hydrogels, Figure 3b,c.

Drying the S‐PEDOT Re‐SPN took 18 h at room temperature (22 °C). In comparison, the swelling rate was rapid, with a water re‐gain of 84% after just 1 min and 158% after 3 min. If left in water, the S‐PEDOT Re‐SPN could reswell to considerably above its original water content coming to an equilibrium at 217% water re‐gain, compared to 42% for the PEDOT:PSS SPN and 17% for S‐PEDOT SPN, Figure 3d. Water re‐gain is quantified as the amount of water gained by the hydrogel during swelling, as a percentage of the initial water content in the hydrogel before drying. The extent of swelling for the S‐PEDOT Re‐SPN can therefore easily be controlled by the volume of immersion water. The conductivity after reswelling stayed relatively constant and still above that of the PEDOT:PSS SPN, Figure 3e. To the best of our knowledge, this is the first fully dryable and reswellable conductive hydrogel, although previous reports include partially reswellable devices,^[^ ^51^ ^]^ or reswellable hydrogels with limited conductivity (0.002 S cm^−1^).^[^ ^46^ ^]^ The homogenous conducting network offers resilience against large volumetric and structural changes caused by drying and swelling, while inhomogeneous percolative networks are unlikely to reconstruct original conductive pathways.

The rate of drying for the S‐PEDOT SPNs was slower compared to the PEDOT:PSS SPN, with a drying half time (the time taken to loose half of the hydrogels water) of 4.3 h for the S‐PEDOT SPN and 4.2 h for the S‐PEDOT Re‐SPN, compared to 3.1 h for the PEDOT:PSS SPN, Figure 3f. Slower drying times may be attributed to improved network formation of S‐PEDOT, increasing the total interaction between water molecules and the charged polymer network.^[^ ^52^ ^]^ Interestingly, during the drying period, the extent of increase in conductivity was higher for the PEDOT:PSS network, possibly on account of percolation effects and improved connectivity, although it still remained lower than the S‐PEDOT SPN, Figure S9 (Supporting Information).

## 4 Bioelectronic Applicability

The bioelectronic applicability was initially investigated through skin impedance measurements, important for wearable bioelectronic signal monitoring, Figure 3h. The conductive hydrogels were patterned to match the active area dimensions of commercially available Kendall EMG/ECG electrodes, and placed on the inside of the forearm alongside a counter and reference electrode, Figure S10 (Supporting Information). The S‐PEDOT SPN showed significantly lower impedance overall, especially at frequencies below 50 Hz with an impedance of 1 × 10^4^ Ω compared to 5 × 10^4^ Ω for the PEDOT:PSS SPN and 10^5^ ‐ 10^6^ Ω for the commercially available electrode. The reduced impedance may come from a higher concentration of mobile ions on account of more sulfonic acid groups in the S‐PEDOT SPNs than PEDOT:PSS SPNs. The S‐PEDOT SPNs were subsequently shown to effectively measure biological signals including surface EMG and ECG, as well in the fabrication of wearable LED circuits, Figure S11 (Supporting Information).

Taking advantage of the softness, stretchability and conductivity of the S‐PEDOT SPN, we demonstrated the material as a wearable strain sensor for bioelectronic applications, Figure 3g. The strain sensor was fabricated by patterning the S‐PEDOT SPN through laser cutting, following which the patterned materials was attached to a SPN substrate. The S‐PEDOT SPN adheres to the underlying substrate layer on account of dynamic cross‐link formation at the interface, Figure S12 (Supporting Information), attributed to the high‐binding affinity supramolecular complexes used in both materials.^[^ ^53^ ^]^ Placed over the joint of the index finger, the sensor measured bending through real time resistance change. No delamination was observed throughout the measurements, likely on account of hydrogen bonding between the SPN and skin, showcasing suitability toward on‐skin applications.

The S‐PEDOT SPN further outperformed the PEDOT:PSS SPN in charge injection capacitance ability, indicative of the electrical stimulation efficacy for implantable bioelectronic devices. The application of a biphasic voltage pulse at 200 ms of ±0.5 V resulted in the S‐PEDOT SPN giving a charge injection capacitance of 1.41 mC cm^−2^, compared to 0.45 mC cm^−2^ for the PEDOT:PSS SPN and 0.27 mC cm^−2^ for a gold control electrode, Figures 3i,j. The improved performance can be attributed to an increase in effective volumetric capacitance on account of greater aerial contact of conductive chains with the electrolyte in the homogeneous network compared to the aggregated network.^[^ ^54^ ^]^

## 5 Conclusion

We demonstrate homogeneous integration of hydrophilic self‐doped conductive polymers with durable and functional CB[8] mediated supramolecular hydrogels. Compared with traditionally used PEDOT:PSS, the S‐PEDOT based hydrogel achieved an order of magnitude higher conductivity while maintaining the exemplary mechanical properties of supramolecular networks. The uniform ion‐electron mixed transport enables enhanced charge injection capacitance and improved skin‐impedance performance. Further molecular tailoring of conductive polymers to increase hydrophilicity and the degree of self‐doping may lead to greater milestones in mixed ion‐electron transport ability and mechanical property performance. Our materials design diversifies the function of conductive hydrogels, which is exemplified by the fabrication of dryable and reswellable conductive networks. Potential additional functionalities can include biodegradability, recyclability, cell‐scaffolding, reduced foreign body response, and programmed drug delivery, offering promising new directions in the field of bioelectronics.

## 6 Experimental Section

### Materials

Unless otherwise stated, all the chemicals used in the present work were purchased from Sigma–Aldrich and used directly without further purification: acrylamide (for molecular biology, 99%, high‐performance liquid chromatography (HPLC)), 2‐Acrylamido 2‐dimethylethanesulfonic acid (AMPS, 99%) 1‐vinylimidazole (99%), α, α' ‐Dichloro‐o‐xylene (98%), pyridine (99.8%), acetonitrile (HPLC, 99.9%), diethyl ether (ACS reagent, 99%), ethanol (absolute, 99.8%, HPLC), deuterium oxide (D_2_O, D 99.8%), glycerol (ACS reagent, 99.5%), *N,N*'‐dimethyl acrylamide (DMA, 99%, purified through an alumina plug to remove monomethyl ether hydroquinone inhibitor), nitrogen, 2‐hydroxy‐4‐(2‐hydroxyethoxy)‐2‐methylpropiophenone (photoinitiator, I‐2959, 98%), Hydroxymethyl EDOT (HMEDOT, LEAP Chem, 97%), 2,4‐buthanesultone (LEAP Chem, 98%), iron(III) *p*‐toluenesulfonate hexahydrate (FeTOS, technical grade), Iron(II) sulfate heptahydrate (99%), ammonium persulfate (APS, 98%), *N,N,N',N'*‐Tetramethyl ethylenediamine (TEMED, 99%), 3,4‐Ethylenedioxythiophene (EDOT, 97%), Poly(4‐styrenesulfonic acid) solution (PSS, Mw ≈75,000, 18 wt.% in H_2_O). Cucurbit[8]uril (CB[8]) was prepared and purified using a previously reported method.^[^ ^55^, ^56^ ^]^ Milli‐Q water was simply obtained from a Milli‐Q Integral Water Purification System (18.2 MΩ ·cm). Unless otherwise noted, all the sample solutions were prepared in D_2_O or Milli‐Q H_2_O under heating and ultrasonication.

### Synthesis of Self Doped PEDOT Supramolecular Polymer Networks (S‐PEDOT SPN)

Initially, acrylamide monomer (4 M), photoinitiator (I‐2959, 0.03 Mol.% compared to acrylamide) and the non‐covalent crosslinker 2BPyVI‐CB[8] (2.5 Mol.% compared to acrylamide) were weighed out in a glass vial and dissolved in Milli‐Q water with the precalculated volume under ultrasonication 10 min. The solution was purged with nitrogen for 30 min to remove residual oxygen in the solution phase, which could eliminate radicals during the polymerization. The precursor solution was injected into a laboratory‐made glass mould until the mould was filled. The glass mould filled with the precursor solution was exposed to ultraviolet irradiation at 350 nm for 6 h to undergo photopolymerization. Following the SPN formation, the samples were dried overnight and then immersed in a sulfuric acid solution (1 M) with certain amounts of S‐EDOT and Iron(II) sulfate (0.6 equiv.) at 4 °C for 30 min. Following this, ammonium persulfate solution (0.9 gml^−1^) was added dropwise at 0.1 mlmin^−1^ for 10 min while shaking at 0 °C. The SPN was left 4 °C for 2 days, following which they were left at room temperature for another 2 days to ensure maximum polymerization of S‐PEDOT while minimizing evaporation of solvent. The SPNs were then washed three times with 2M sulfuric acid solution to remove unreacted S‐EDOT and iron(III) *p*‐toluenesulfonate hexahydrate, following which the E‐SPNs were washed three times with water to replace the sulfuric acid. The same procedure was used for S‐PEDOT RE‐SPN, however starting with acrylamide (3 M) and 2‐Acrylamido 2‐dimethylethanesulfonic acid (3 M) as the strating monomer composition. The PEDOT:PSS SPN was synthesized as previously reported.^[^ ^29^ ^]^ The SPN materials were patterned for electrodes or sensors using a nanosecond UV (wavelength: 355 nm) laser marker (Keyence, MD‐U1000C). A power of 80% was used and 40 iterations were performed to ensure complete patterning of samples up to 1 mm thickness.

### Fluorescence Microscopy

Samples were placed on glass slides (VWR Cat No. 631 – 0124, 22 mm x 22 mm, Thickness No. 1) and imaged using a Nikon Eclipse Ti2 inverted microscope with 10x objective (zoomed out), 20x air objective (mid‐zoom), and a 100x oil (NA = 1.49) immersion objective (zoomed‐in features). Samples were illuminated at 488 nm (LuxXplus 488–200 diode laser) with approximately 20–30 mW output power (10/20x objectives, 30% current) or <5 mW output power (100x objective, 1% current; power was decreased until image features were not saturated) in epifluorescence mode. A dichroic quadpass cube (405, 488, 561, and 640 nm) was used to allow the laser to pass for excitation and a longpass emission filter (>488 nm) was utilized in the microscope to block the laser excitation before the detector. Images were recorded on a Photometrics Prime BSI sCMOS camera with 10–30 ms (10/20x objectives) and 5 ms (100x objective) integration times.

### Rheology

Rheological characterization was carried out by a Discovery Hybrid Rheometer (DHR)‐2, TA Instruments, with a Peltier Plate for temperature control. All the measurements were conducted using a 20 mm parallel stainless steel plate geometry, and the necessary calibration for geometry was carried out before testing. All the samples for rheological characterization were measured just after the SPN fabrication. Oscillatory frequency‐sweep measurements were conducted at 1% strain in the frequency range from 0.1 to 100 rad s^−1^. Continuous step‐strain measurements were performed at 1.0 rad s^−1^ with an amplitude oscillation of 1% strain. The data was collected at 293.15 K and analyzed by TRIOS software, TA Instruments.

### Mechanical Testing

Tensile tests were performed, just after the network synthesis, on a Tinius Olsen Model HK 25‐kN Benchtop Tester machine equipped with a 25‐N load cell at room temperature. A typical tensile test was performed by stretching a dumbbell‐shaped specimen (following ISO4661‐1 standard) at 50 mm min^−1^ until its breakage, to obtain stress‐strain curves. Young's modulus and toughness are calculated from the slope and the area under the curve, respectively.

### Electrical Characterization

To measure electrical conductivity, a four‐point probe method was employed, using a Keithley 2400 source meter to apply a set voltage and record the current. To measure impedance, potentiostatic electrochemical impedance spectroscopy was recorded using a PalmSens (PalmSens4) potentiostat. The SPN specimens were placed between two silver‐ink based electrodes and AC impedance measurements were obtained between 100 kHz to 500 mHz with an applied amplitude of 20 mV_*rms*_ relative to the open circuit potential. To measure the electrical properties during deformation, an LCR meter (NF Corporation, ZM2376) was used to measure the impedance across the stretched E‐SPN sample at a fixed frequency of 1 kHz, while the E‐SPN was stretched up to 100% strain. To measure charge injection capacity (CIC), biphasic pulses at 200 ms with an amplitude of ±0.5 V were applied by using a PalmSens (PalmSens4) potentiostat with the sample as a working electrode, an Ag/AgCl wire as a reference electrode, a platinum coil as a counter electrode, and PBS as an electrolyte.

### Cryo Scanning Electron Microscopy

Cryo‐SEM was performed using a Zeiss Crossbeam 550 (Carl Zeiss, Oberkochen, Germany) focused ion beam scanning electron microscope (FIB‐SEM) at an accelerating voltage of 2 kV. The samples were maintained at cryogenic temperatures of −170 °C using a Quorum 3010 Cryo stage (Quorum Technologies, Loughton, UK). The samples were frozen on high‐pressure freezer planchettes using a LEICA MM80 Metal Mirror freezer to ensure vitreous freezing. After freezing they were secured to the cryo‐SEM shuttle and the cryo‐sledge with the samples was transferred to the preparation chamber. Samples were sputter coated with Pt at a current of 10 mA for 60s. Samples were transferred to the main stage of the microscope and imaged using a range of detection modes including the SESI and inLens detectors at 2kV accelerating voltage. Standard imaging magnifications were used to allow comparison of different samples. Prior to the Cryo‐FIB cross‐sectioning, a layer of organometallic precursor was deposited from the gas injector system (GIS) for approx. 45–60 s.

### In vivo Recordings

All experiments were performed with the approval of the Ethics Committee of the Department of Engineering at the University of Cambridge (6/9/2018, IONBIKE), and with consent from the volunteers. Before the electrodes were placed on the arm of the volunteer, the underlying skin was gently wiped with a tissue soaked in ethanol. The electrodes were left on the skin for 5 min before measurements were taken to ensure stabilization of the electrode/skin interface. For the impedance measurements, a PalmSens (PalmSens4) potentiostat was used, with two commercial Kendall electrodes as reference and counter electrodes, which were also placed on the skin. For the EMG recordings, both the E‐SPN and commercial Kendall electrodes were recorded simultaneously on the skin during forearm and grip contractions using a RHS stimulation and recording system from Intan technologies, with a reference electrode placed on the elbow. The sampling rate was 30 kHz. The acquired signals were filtered using a 50 Hz band‐stop filter, and a band pass filter with cut off frequencies of 10 and 200 Hz. For ECG recordings, the S‐PEDOT SPN was placed on the wrist and the signals were preprocessed by applying notch filtering at 50 Hz and bandpass filtering between 0.1 and 100 Hz.

## Conflict of Interest

The authors declare no conflict of interest.

## Supporting information

Supporting Information

## Data Availability

The data that support the findings of this study are available from the corresponding author upon reasonable request.

## References

[1] T. Someya , Z. Bao , G. G. Malliaras , Nature 2016, 540, 379. 27974769 10.1038/nature21004

[2] E. Song , J. Li , S. M. Won , W. Bai , J. A. Rogers , Nat. Mater. 2020, 19, 590. 32461684 10.1038/s41563-020-0679-7

[3] H. Yuk , B. Lu , X. Zhao , Chem. Soc. Rev. 2019, 48, 1642. 30474663 10.1039/c8cs00595h

[4] J. Deng , H. Yuk , J. Wu , C. E. Varela , X. Chen , E. T. Roche , C. F. Guo , X. Zhao , Nat. Mater. 2021, 20, 229. 32989277 10.1038/s41563-020-00814-2

[5] Z. Huang , Y. Hao , Y. Li , H. Hu , C. Wang , A. Nomoto , T. Pan , Y. Gu , Y. Chen , T. Zhang , W. Li , Y. Lei , N. Kim , C. Wang , L. Zhang , J. W. Ward , A. Maralani , X. Li , M. F. Durstock , A. Pisano , Y. Lin , S. Xu , Nat. Electron. 2018, 1, 473.

[6] H. Yuk , J. Wu , X. Zhao , Nat. Rev. Mater. 2022, 7, 935.

[7] S.‐H. Sunwoo , S. I. Han , C. S. Park , J. H. Kim , J. S. Georgiou , S.‐P. Lee , D.‐H. Kim , T. Hyeon , Nat. Rev. Bioeng. 2023, 1, 17.

[8] Y. Ohm , C. Pan , M. J. Ford , X. Huang , J. Liao , C. Majidi , Nat. Electron. 2021, 4, 185.

[9] J. Rivnay , R. M. Owens , G. G. Malliaras , Chem. Mater. 2014, 26, 679.

[10] J. Rivnay , S. Inal , A. Salleo , R. M. Owens , M. Berggren , G. G. Malliaras , Nat. Rev. Mater. 2018, 3, 1.

[11] C. Yang , Z. Suo , Nat. Rev. Mater. 2018, 3, 125.

[12] C. M. Tringides , N. Vachicouras , I. de Lázaro , H. Wang , A. Trouillet , B. R. Seo , A. Elosegui‐Artola , F. Fallegger , Y. Shin , C. Casiraghi , K. Kostarelos , S. P. Lacour , D. J. Mooney , Nat. Nanotechnol. 2021, 16, 1019. 34140673 10.1038/s41565-021-00926-z PMC9233755

[13] C. Keplinger , J.‐Y. Sun , C. C. Foo , P. Rothemund , G. M. Whitesides , Z. Suo , Science 2013, 341, 984. 23990555 10.1126/science.1240228

[14] J.‐Y. Sun , X. Zhao , W. R. Illeperuma , O. Chaudhuri , K. H. Oh , D. J. Mooney , J. J. Vlassak , Z. Suo , Nature 2012, 489, 133. 22955625 10.1038/nature11409 PMC3642868

[15] Z. Huang , X. Chen , S. J. O'Neill , G. Wu , D. J. Whitaker , J. Li , J. A. McCune , O. A. Scherman , Nat. Mater. 2022, 21, 103. 34819661 10.1038/s41563-021-01124-x

[16] M. J. Webber , E. A. Appel , E. Meijer , R. Langer , Nat. Mater. 2016, 15, 13. 26681596 10.1038/nmat4474

[17] M. J. Webber , M. W. Tibbitt , Nat. Rev. Mater. 2022, 7, 541.

[18] B. Lu , H. Yuk , S. Lin , N. Jian , K. Qu , J. Xu , X. Zhao , Nat. Commun. 2019, 10, 1. 30837483 10.1038/s41467-019-09003-5 PMC6401010

[19] R. Green , M. R. Abidian , Adv. Mater. 2015, 27, 7620. 26414302 10.1002/adma.201501810 PMC4681501

[20] Y. Liu , J. Liu , S. Chen , T. Lei , Y. Kim , S. Niu , H. Wang , X. Wang , A. M. Foudeh , J. B.‐H. Tok , Z. Bao , Nat. Biomed. Eng. 2019, 3, 58. 30932073 10.1038/s41551-018-0335-6

[21] V. R. Feig , H. Tran , M. Lee , Z. Bao , Nat. Commun. 2018, 9, 1. 30013027 10.1038/s41467-018-05222-4 PMC6048132

[22] S. Zhang , Y. Chen , H. Liu , Z. Wang , H. Ling , C. Wang , J. Ni , B. Çelebi‐Saltik , X. Wang , X. Meng , H.‐J. Kim , A. Baidya , S. Ahadian , N. Ashammakhi , M. R. Dokmeci , J. Travas‐Sejdic , A. Khademhosseini , Adv. Mater. 2020, 32, 1904752. 10.1002/adma.201904752 PMC6946856 31657081

[23] T. Zhou , H. Yuk , F. Hu , J. Wu , F. Tian , H. Roh , Z. Shen , G. Gu , J. Xu , B. Lu , X. Zhao , Nat. Mater. 2023, 22, 1. 37322141 10.1038/s41563-023-01569-2

[24] B. D. Paulsen , K. Tybrandt , E. Stavrinidou , J. Rivnay , Nat. Mater. 2020, 19, 13. 31427743 10.1038/s41563-019-0435-z

[25] J. Chong , C. Sung , K. S. Nam , T. Kang , H. Kim , H. Lee , H. Park , S. Park , J. Kang , Nat. Commun. 2023, 14, 2206. 37072411 10.1038/s41467-023-37948-1 PMC10113367

[26] S. Inal , G. G. Malliaras , J. Rivnay , Nat. Commun. 2017, 8, 1767. 29176599 10.1038/s41467-017-01812-w PMC5701155

[27] Y. Shin , H. S. Lee , Y. J. Hong , S.‐H. Sunwoo , O. K. Park , S. H. Choi , D.‐H. Kim , S. Lee , Sci. Adv. 2024, 10, eadi7724. 38507496 10.1126/sciadv.adi7724 PMC10954228

[28] H. Yano , K. Kudo , K. Marumo , H. Okuzaki , Sci. Adv. 2019, 5, 9492. 10.1126/sciadv.aav9492 PMC6461456 30993206

[29] S. J. O'Neill , Z. Huang , M. H. Ahmed , A. J. Boys , S. Velasco‐Bosom , J. Li , R. M. Owens , J. A. McCune , G. G. Malliaras , O. A. Scherman , Adv. Mater. 2023, 35, 2207634. 10.1002/adma.202207634 36314408

[30] K. M. Persson , R. Karlsson , K. Svennersten , S. Löffler , E. W. Jager , A. Richter‐Dahlfors , P. Konradsson , M. Berggren , Adv. Mater. 2011, 23, 4403. 21960476 10.1002/adma.201101724

[31] R. H. Karlsson , A. Herland , M. Hamedi , J. A. Wigenius , A. Åslund , X. Liu , M. Fahlman , O. Inganas , P. Konradsson , Chem. Mater. 2009, 21, 1815.

[32] I. Ivanko , J. Pánek , J. Svoboda , A. Zhigunov , E. Tomšík , J. Mater. Chem. C 2019, 7, 7013.

[33] J. L. Davis , B. Dong , C. Sun , H. F. Zhang , J. Biomed. Opt. 2018, 23, 106501. 30334394 10.1117/1.JBO.23.10.106501 PMC6210800

[34] R. Noriega , J. Rivnay , K. Vandewal , F. P. Koch , N. Stingelin , P. Smith , M. F. Toney , A. Salleo , Nat. Mater. 2013, 12, 1038. 23913173 10.1038/nmat3722

[35] D. Miklavčič , N. Pavšelj , F. X. Hart , Wiley encyclopedia of biomedical engineering, Wiley, New York 2006.

[36] D. Dean , T. Ramanathan , D. Machado , R. Sundararajan , J. Electrost. 2008, 66, 165. 10.1016/j.elstat.2007.11.005 PMC2597841 19255614

[37] C. F. Guimarães , L. Gasperini , A. P. Marques , R. L. Reis , Nat. Rev. Mater. 2020, 5, 351.

[38] A. Arani , S. P. Arunachalam , I. C. Chang , F. Baffour , P. J. Rossman , K. J. Glaser , J. D. Trzasko , K. P. McGee , A. Manduca , M. Grogan , A. Dispenzieri , R. L. Ehman , P. A. Araoz , J. Magn. Reson. Imaging 2017, 46, 1361. 28236336 10.1002/jmri.25678 PMC5572539

[39] W. Xu , Q. Song , J.‐F. Xu , M. J. Serpe , X. Zhang , ACS Appl. Mater. Interfaces 2017, 9, 11368. 28322541 10.1021/acsami.7b02850

[40] Z. Huang , X. Chen , G. Wu , P. Metrangolo , D. Whitaker , J. A. McCune , O. A. Scherman , J. Am. Chem. Soc. 2020, 142, 7356. 32248683 10.1021/jacs.0c02275 PMC7181256

[41] E. A. Appel , J. del Barrio , X. J. Loh , O. A. Scherman , Chem. Soc. Rev. 2012, 41, 6195. 22890548 10.1039/c2cs35264h

[42] L. Voorhaar , R. Hoogenboom , Chem. Soc. Rev. 2016, 45, 4013. 27206244 10.1039/c6cs00130k

[43] O. Chaudhuri , L. Gu , D. Klumpers , M. Darnell , S. A. Bencherif , J. C. Weaver , N. Huebsch , H.‐p. Lee , E. Lippens , G. N. Duda , D. J. Mooney , Nat. Mater. 2016, 15, 326. 26618884 10.1038/nmat4489 PMC4767627

[44] D. Xia , P. Wang , X. Ji , N. M. Khashab , J. L. Sessler , F. Huang , Chem. Rev. 2020, 120, 6070. 32426970 10.1021/acs.chemrev.9b00839

[45] Y. Lee , J. W. Chung , G. H. Lee , H. Kang , J.‐Y. Kim , C. Bae , H. Yoo , S. Jeong , H. Cho , S.‐G. Kang , J. Y. Jung , D. W. Lee , S. Gam , S. G. Hahm , Y. Kuzumoto , S. J. Kim , Z. Bao , Y. Hong , Y. Yun , S. Kim , Sci. Adv. 2021, 7, eabg9180. 34088675 10.1126/sciadv.abg9180 PMC8177712

[46] M. Zhao , W. Zhang , P. Sun , L. Shi , Y. Liu , X. Zhang , L. Zhang , W. Han , P. Chen , ACS Sustain. Chem. Eng. 2022, 10, 6657.

[47] J. Huang , Z. Zhang , H. Jiang , Appl. Mater. Today 2023, 32, 101786.

[48] X. Liu , C. Steiger , S. Lin , G. A. Parada , J. Liu , H. F. Chan , H. Yuk , N. V. Phan , J. Collins , S. Tamang , G. Traverso , X. Zhao , Nat. Commun. 2019, 10, 493. 30700712 10.1038/s41467-019-08355-2 PMC6353937

[49] A. H. Karoyo , L. D. Wilson , Materials 2021, 14, 1095.33652859

[50] S. K. De , N. Aluru , B. Johnson , W. Crone , D. J. Beebe , J. Moore , J. Microelectromech. Syst. 2002, 11, 544.

[51] M. Sasaki , B. C. Karikkineth , K. Nagamine , H. Kaji , K. Torimitsu , M. Nishizawa , Adv. Healthcare Mater. 2014, 3, 1919. 10.1002/adhm.201400209 24912988

[52] Y. Wang , P. Chen , X. Zhou , Y. Liu , N. Wang , C. Gao , ACS Appl. Mater. Interfaces 2022, 14, 47100. 36194533 10.1021/acsami.2c14157

[53] S. J. K. O'Neill , Z. Huang , X. Chen , R. L. Sala , J. A. McCune , G. G. Malliaras , O. A. Scherman , Sci. Adv. 2024, 10, eadn5142. 39018406 10.1126/sciadv.adn5142 PMC466958

[54] N. A. Kukhta , A. Marks , C. K. Luscombe , Chem. Rev. 2021, 122, 4325. 34902244 10.1021/acs.chemrev.1c00266 PMC8874907

[55] J. Kim , I.‐S. Jung , S.‐Y. Kim , E. Lee , J.‐K. Kang , S. Sakamoto , K. Yamaguchi , K. Kim , J. Am. Chem. Soc. 2000, 122, 540.

[56] A. Day , A. P. Arnold , R. J. Blanch , B. Snushall , J. Org. Chem. 2001, 66, 8094. 11722210 10.1021/jo015897c

