# Supplementary material for: Supramolecular Conductive Hydrogels With Homogeneous Ionic and Electronic Transport
Source: Adv Mater. 2025 Apr 28;37(26):2415687. doi: 10.1002/adma.202415687 (PMC12232228; doi:10.1002/adma.202415687)
Supplement: Supplementary file 1 — Supporting Information [file ADMA-37-2415687-s001.pdf]

ADVANCED MATERIALS

Supporting Information

for *Adv. Mater.*, DOI 10.1002/adma.202415687

Supramolecular Conductive Hydrogels With Homogeneous Ionic and Electronic Transport

Stephen J.K. O'Neill, Minoru Ashizawa, Alan M. McLean, Ruben Ruiz-Mateos Serrano, Tokihiko Shimura, Masakazu Agetsuma, Motosuke Tsutsumi, Tomomi Nemoto, Christopher D. J. Parmenter, Jade A. McCune, George G. Malliaras, Naoji Matsuhisa and Oren A. Scherman**

Supplementary Information: Supramolecular conductive hydrogels with homogeneous ionic and electronic transport

Stephen J.K. O'Neill[1], Minoru Ashizawa[2], Alan M. McLean[1], Ruben Ruiz-Mateos Serrano[3], Tokihiko Shimura[4,5,6], Masakazu Agetsuma[7,8], Motosuke Tsutsumi[7,8], Tomomi Nemoto[9,10], Christopher D. J. Parmenter[11], Jade A. McCune[1], George G. Malliaras[3], Naoji Matsuhisa*[4, 5, 6], and Oren A. Scherman*[1]

[1] *Melville Laboratory for Polymer Synthesis, Yusuf Hamied Department of Chemistry, University of Cambridge, Lensfield Road, Cambridge CB2 1EW, UK.*

[2] *Department of Materials Science and Engineering, Tokyo Institute of Technology, 2-12-1 Ookayama, Meguro-ku, Tokyo, 152-8552, Japan.*

[3] *Electrical Engineering Division, Department of Engineering, University of Cambridge, 9 JJ Thomson Ave, Cambridge, CB3 0FA, U.K.*

[4] *Research Center for Advanced Science and Technology, The University of Tokyo, 4-6-1 Komaba, Meguro-ku, Tokyo 153-8505, Japan.*

[5] *Electronics and Electrical Engineering, Faculty of Science and Engineering, Keio University, 3-14-1 Hiyoshi, Kohoku-ku, Yokohama, Kanagawa, 223-8522, Japan.*

[6] *Institute of Industrial Science, The University of Tokyo, 4-6-1 Komaba, Meguro-ku, Tokyo 153-8505, Japan.*

[7] *Division of Homeostatic Development, National Institute for Physiological Sciences, 38 Nishigohnaka Myodaiji-cho, Okazaki, Aichi, 444-8585, Japan.*

[8] *Quantum Regenerative and Biomedical Engineering Team, Institute for Quantum Life Science, National Institutes for Quantum Science and Technology (QST), Anagawa 4-9-1, Chiba Inage-ku, Chiba 263-8555, Japan..*

[9] *Biophotonics Research Group, Exploratory Research Center on Life and Living Systems, National Institutes of Natural Sciences, Okazaki,Aichi, 444-8787 Japan.*
[10] *Research Division of Biophotonics, National Institute for Physiological Sciences, National Institutes of Natural Sciences, Okazaki, Aichi, 444-8787 Japan.*
[11] *Nottingham Nanoscale and Microscale Research Centre, University of Nottingham, University Park, Nottingham, NG7 2RD UK.*
* *e-mail: oas23@cam.ac.uk, naoji@iis.u-tokyo.ac.jp*

S.1 Design & synthesis of guest monomer BPyVI

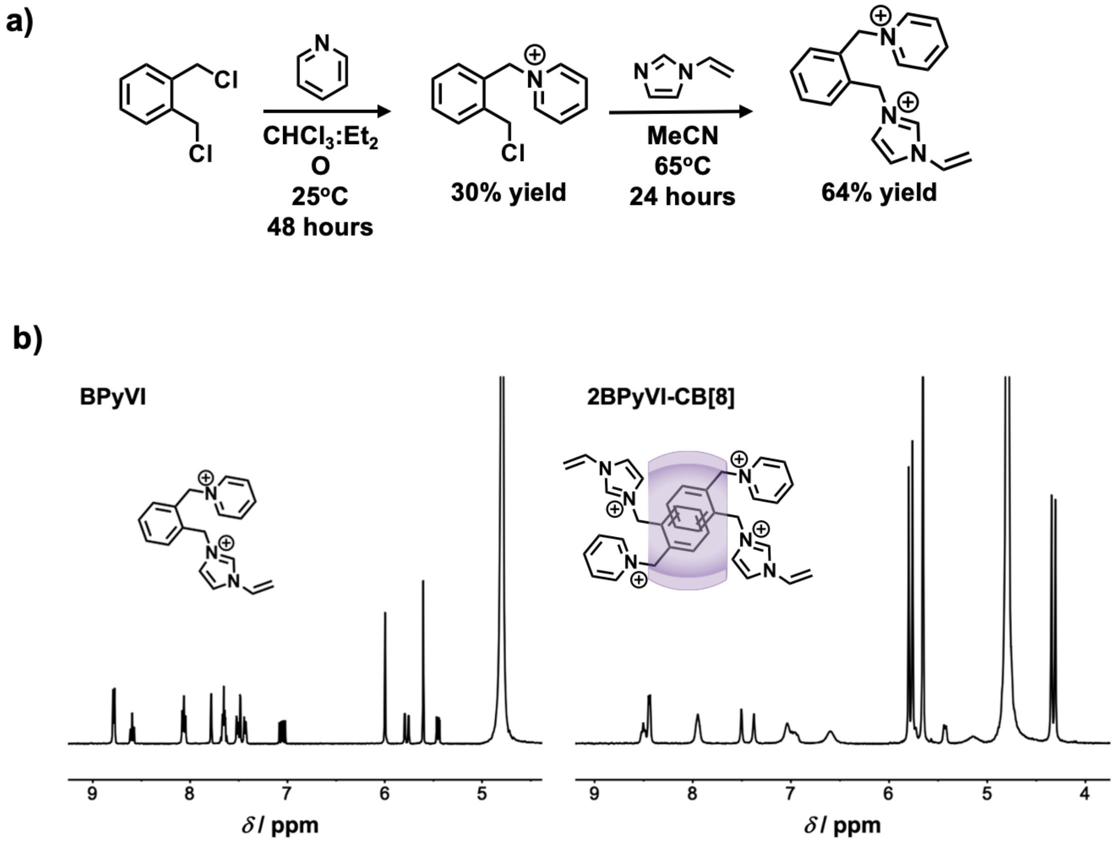

Figure S1: BPyVI guest synthesis. a) Synthetic route for the guest molecule BPyVI, containing two cationic moieties to maximise the ion-dipole interaction between host and guest molecules. b) ^{1}H NMR spectra (D_2O, 298 K) of BPyVI (2 mM, left) and 2BPyVI-CB[8] (1 mM, right). Counterions have been omitted for clarity.

Design of guest monomer BPyVI We recently reported the CB[8] guest molecule 1-(2-((1-vinyl-1H- imidazol-3-ium-3-yl)methyl)benzyl)pyridin-1-ium dichloride (BPyVI) which shows high binding strength to CB[8] ($K_1K_2 = 2.3 \times 10^{13}$ M^{-2}). The two cationic moieties on the BPyVI guest result in a strong ion dipole attraction with the CB[8] portal, alleviating charge screening effects from ionic monomers and polymers, and improving the solubility of the complex. Such characteristics make the crosslink suitable for use with polyions such as PEDOT:PSS.[1]

Synthesis of guest monomer BPyVI BPyVI was synthesised as previously reported.[1] Briefly, α,α'-Dichloro-o-xylene (6.66 g, 38 mmol, 1.0 eq.) and pyridine (3.13 ml, 3.07 g, 38.8 mmol, 1.02 eq.) were added to a mixture of ether (20 ml) and chloroform (5 ml) and refluxed for

48 h. The resulting mixture was precipitated in ether, giving the white solid 1-(2-((1-vinyl-1H-imidazol-3-ium-3-yl)methyl)benzyl)pyridin-1-ium chloride. 1-(2-((1-vinyl-1H-imidazol-3-ium-3-yl)methyl)benzyl)pyridin-1-ium (2.92 g, 11.5 mmol, 1.0 eq., 30% yield) and N-vinylimidazole (2.24 g, 23 mmol, 2.0 eq.) were added to acetonitrile (30 ml) and refluxed overnight. The resulting mixture was again precipitated in ether (30 ml each) resulting in 1-(2-((1-vinyl-1H-imidazol-3-ium-3-yl)methyl)benzyl)pyridin-1-ium dichloride (BPyVI) precipitation as a white solid.

S.2 Synthesis of S-EDOT monomer

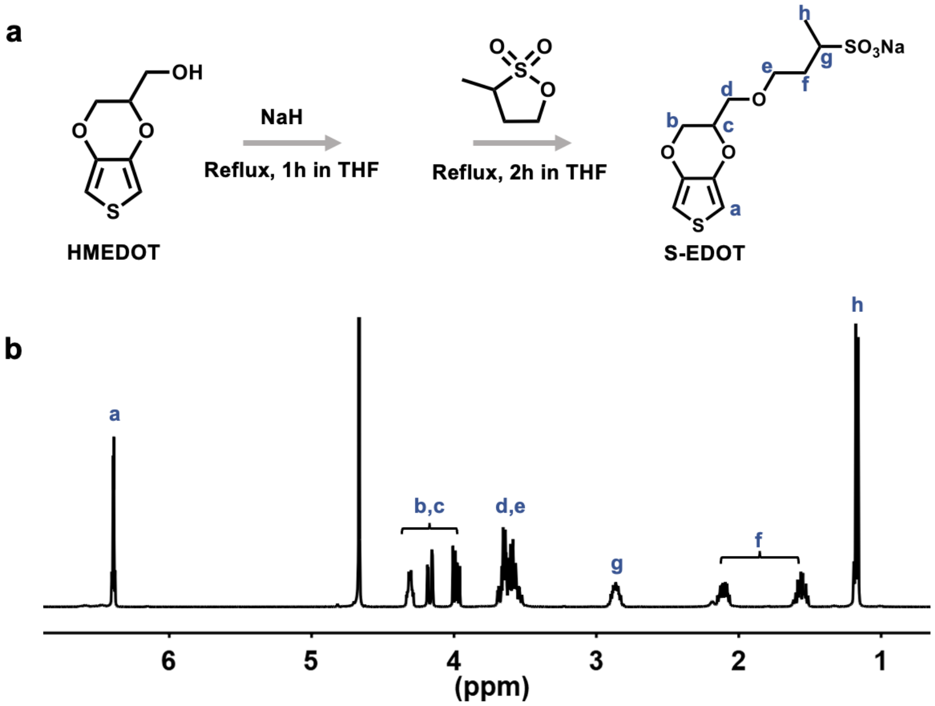

Figure S2: S-EDOT monomer synthesis. a) Synthetic route for the S-EDOT monomer. b) ^{1}H NMR spectra (D_2O, 298 K) of the S-EDOT monomer.

Synthesis of S-EDOT S-EDOT monomer was synthesised as previously reported.[2] Briefly, Hydroxymethyl EDOT (HMEDOT, 2 g, 11.6 mmol) and Sodium hydride (NaH, 0.56 g, 24.3 mmol) were added to THF (40 ml) and refluxed for 1 h. Next, 2,4-butanesultone (1.74 g, 12.8 mmol) dissolved in THF (10 ml) was added dropwise and the mixture was refluxed for 2 h to sulfonate the HMEDOT. After quenching the reaction with EtOH, THF was distilled under reduced pressure, the mixture was dissolved in a minimal amount of acetone and S-EDOT was recrystallized in toluene as a pale yellow solid (92% yield).

S.3 Procedures for preparation of the E-SPN

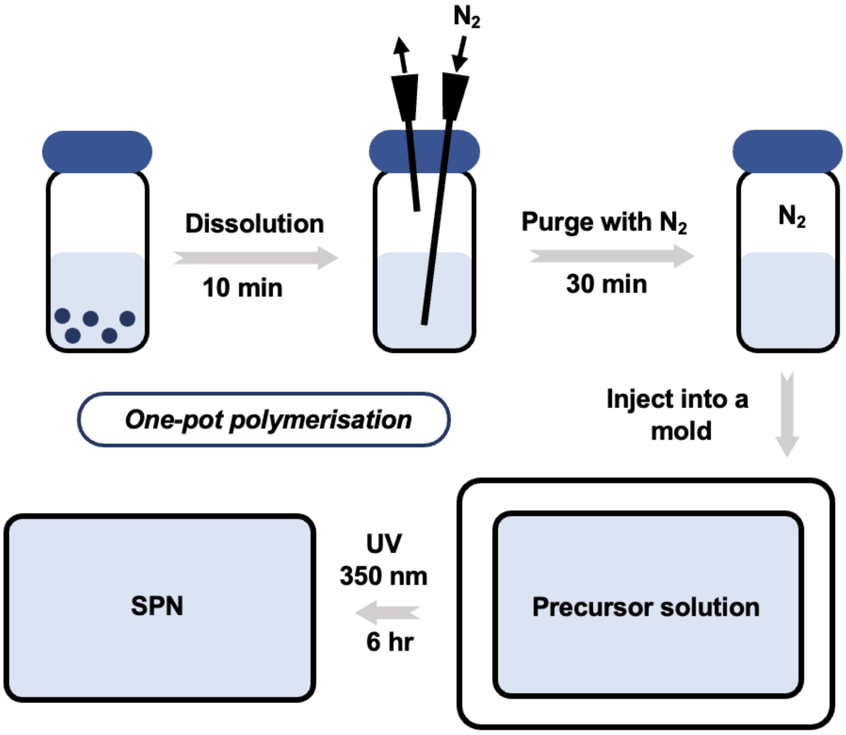

Figure S3: Schematic representation of the fabrication protocol of the SPN. The precursor solution is injected into a polycarbonate mold for free-radical polymerization.

Figure S4 shows the six steps for the preparation of SPNs;

(1) Acrylamide, non-covalent crosslinker (2BPyVI-CB[8]) and initiator were weighed out in a glass vial, and dissolved in pre-calculated amount of Milli-Q water under ultrasonication for 10 min.

(2) The obtained precursor solution was sealed and purged with nitrogen for at least 30 min to remove oxygen in the solution phase that may eliminate radicals during polymerization. The solution was then briefly put under vacuum to remove any dissolved gas, to avoid bubbles in the final hydrogel.

(3) The precursor solution was carefully injected into a laboratory-made, non-stick polycarbonate mold until the whole mold was filled without any bubbles or spare space inside.

(4) The polycarbonate mold, filled with the precursor solution, was then exposed to 350 nm UV for 6 h to undergo photo-polymerization in one pot.

Step-wise E-SPN formation

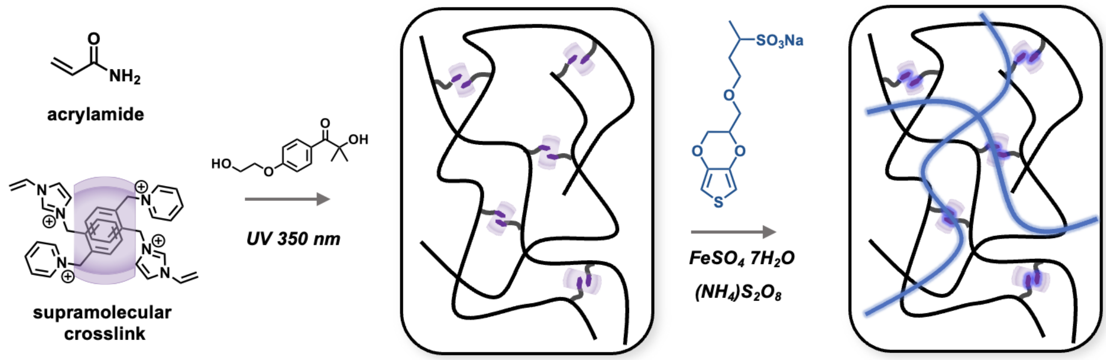

Figure S4: Step-wise synthetic procedure for the formation of the S-PEDOT SPN. The synthetic procedure involves free-radical polymerization of the SPN network through UV photopolymerisation, followed by redox polymerization of S-EDOT for the *in situ* formation of S-PEDOT.

S-EDOT monomer was polymerized within the SPN via redox polymerisation using $(NH_4)_2S_2O_8$ as oxidant and $FeSO_4$ as catalyst to form S-PEDOT. Importantly, S-EDOT is considerably more soluble in water compared to EDOT, enabling high loading in an aqueous environment without the use of organic solvents.

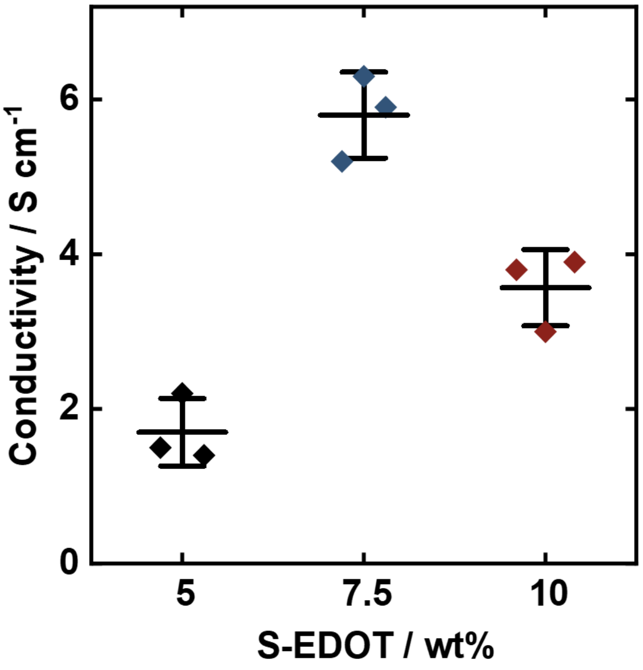

Figure S5: Conductivity of the S-PEDOT SPN. Conductivity comparison of the S-PEDOT SPN prepared at different S-EDOT monomer concentrations for the in-situ polymerisation reaction.

Increasing the concentration of the S-EDOT monomer from 5 wt% to 7.5 wt% significantly increases the conductivity, likely on account of greater conduction pathways for electrons. The lower conductivity at 10 wt% S-EDOT monomer loading has previously been reported and been attributed to lower molecular weights of S-PEDOT at higher monomer concentrations.[2]

S.4 SEM characterization of S-PEDOT SPN

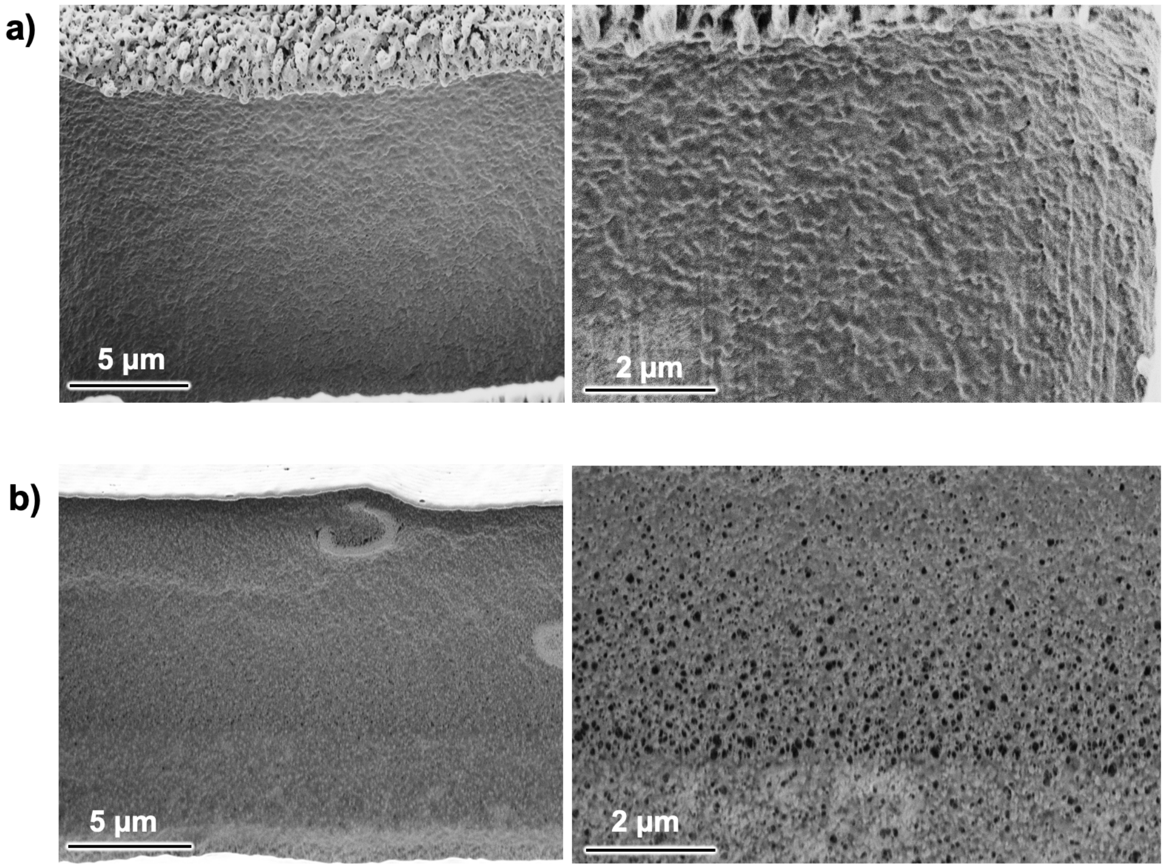

Figure S6: SEM images of the S-PEDOT SPN. a) Cryo-focused ion beam scanning electron micrographs of the S-PEDOT SPN. b) Cryo-focused ion beam scanning electron micrographs of the PEDOT:PSS SPN. Through SEM it is not possible to observe PEDOT distributions across the sample.

S.5 Rheological characterization of S-PEDOT SPN

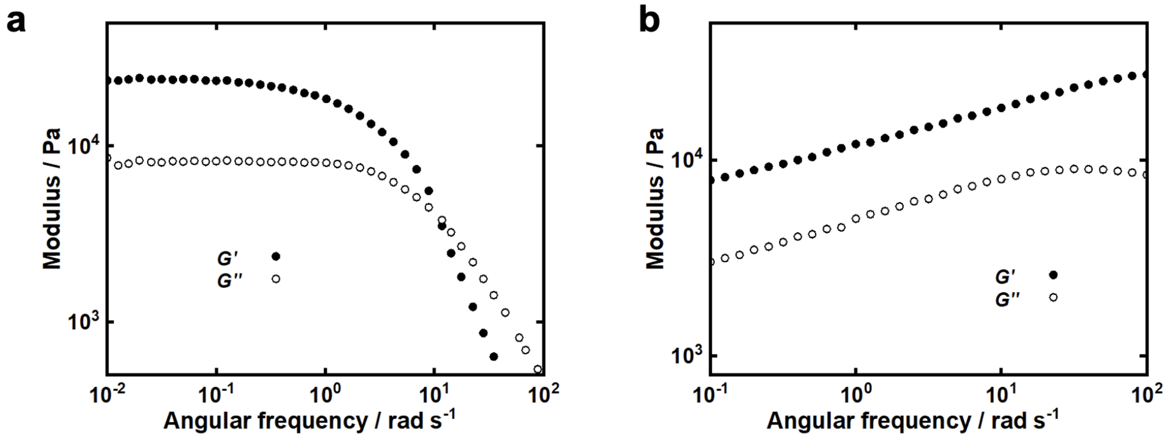

Figure S7: Rheological measurements of the S-PEDOT SPN. a) Rheological strain-sweep measurements of the S-PEDOT SPN. b) Rheological amplitude-sweep measurements of the S-PEDOT SPN.

S.6 Mechanical properties of S-PEDOT Re-SPN

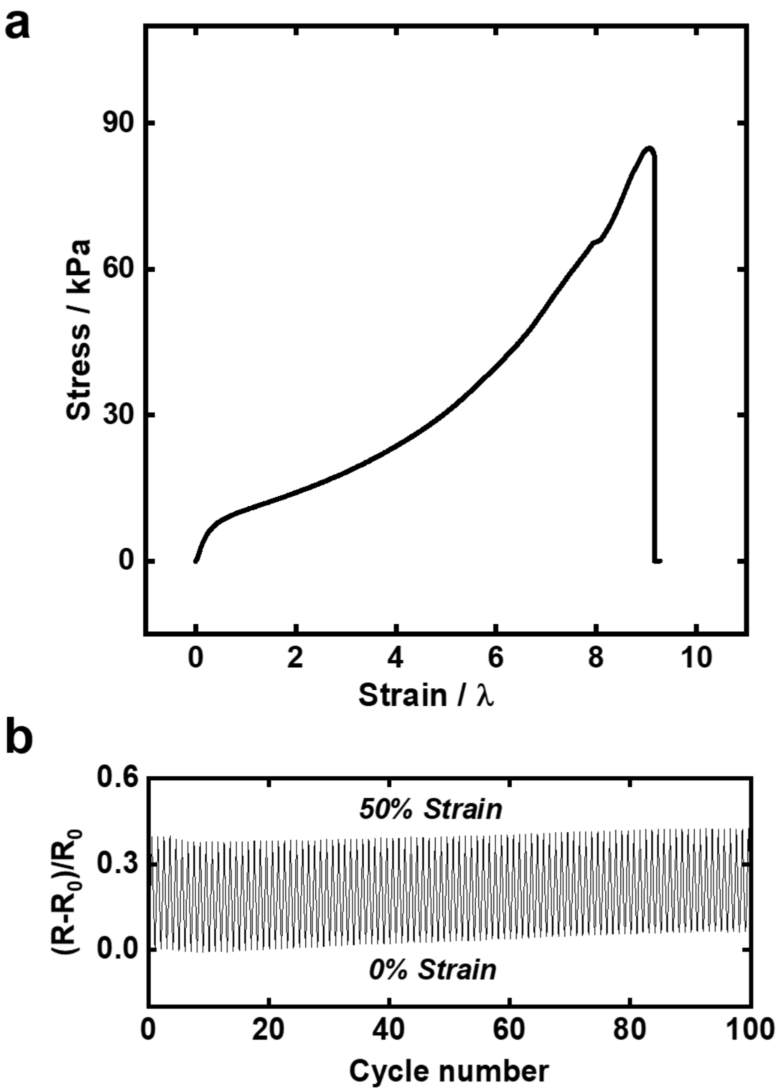

Figure S8: Mechanical properties of S-PEDOT Re-SPN. a) Tensile stress–strain curves of the S-PEDOT Re-SPN. b) Resistance change versus tensile cycle of the S-PEDOT Re-SPN.

S.7 Drying of S-PEDOT SPN

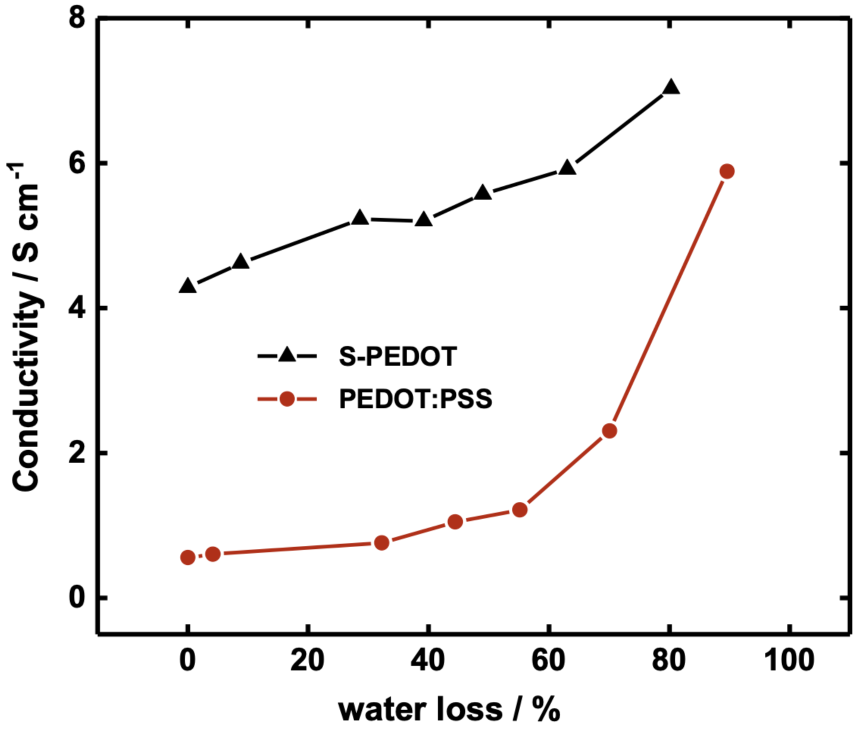

Figure S9: Conductivity of SPN's with drying. a) Plot of conductivity versus relative amount of water loss, measured during the drying of S-PEDOT and PEDOT:PSS SPN's at room temperature.

S.8 Laser Patterning of S-PEDOT SPN

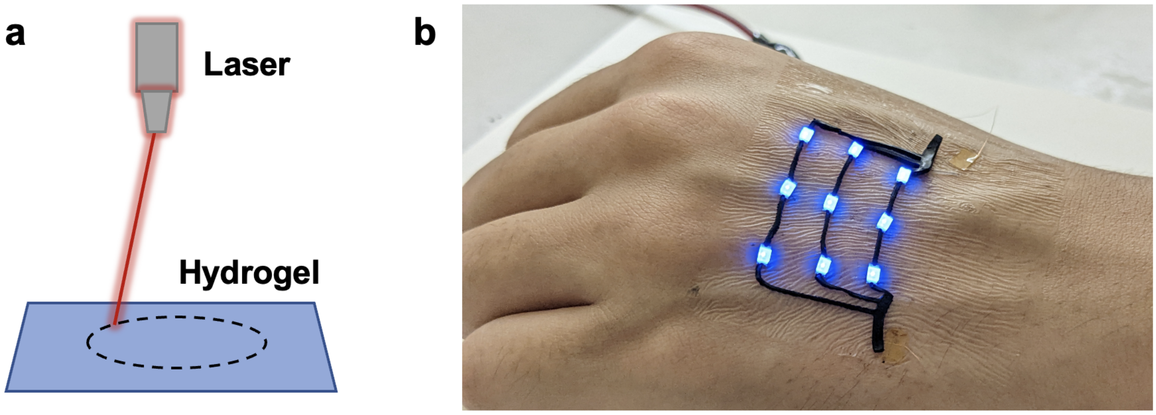

Figure S10: Patterning of S-PEDOT SPNs. a) Schematic of the laser patterning process of the S-PEDOT SPNs. b) A laser patterned S-PEDOT SPN circuit of blue light emitting diodes, demonstrating the potential for wearable displays.

S.9 EMG & ECG Recordings Using S-PEDOT SPN

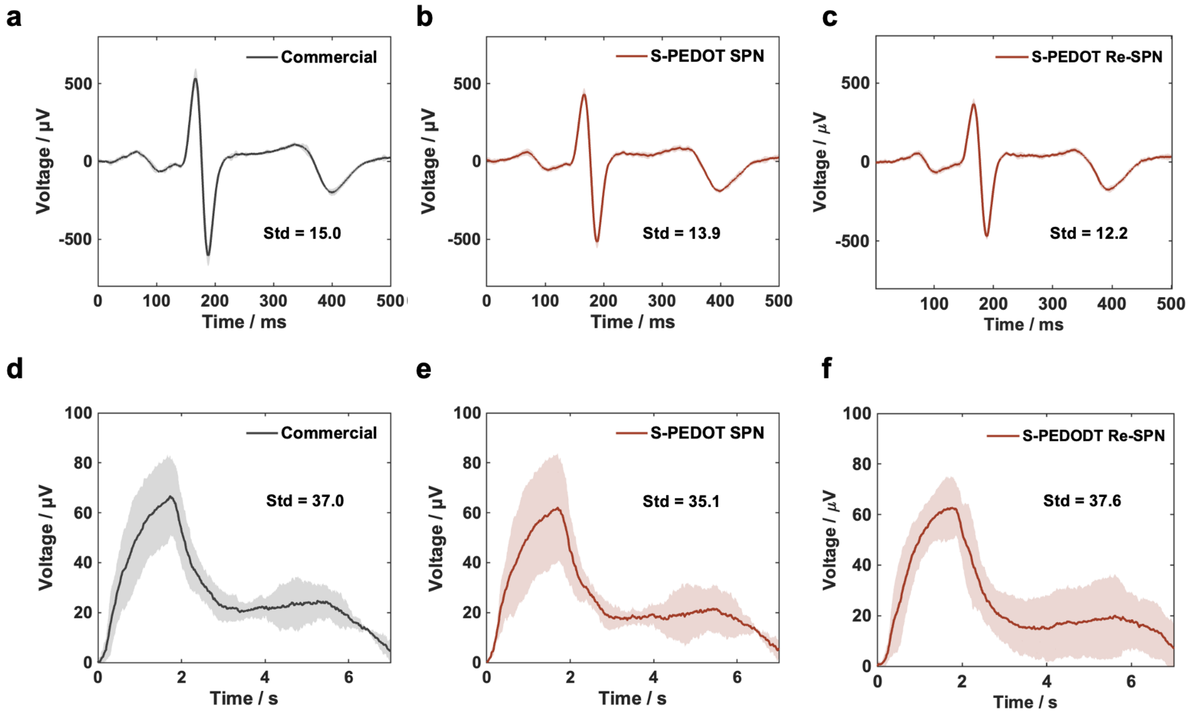

Figure S11: EMG and ECG demonstrations. Time-independent ensemble average of ECG peaks and average standard deviation for: a) commercial electrode, b) S-PEDOT SPN electrode, and, c) A S-PEDOT Re-SPN electrode. Time-independent ensemble average of EMG peaks and average standard deviation of: d) commercial electrode, e) S-PEDOT SPN electrode, f) A S-PEDOT Re-SPN electrode.

S.10 Interfacial Adhesion

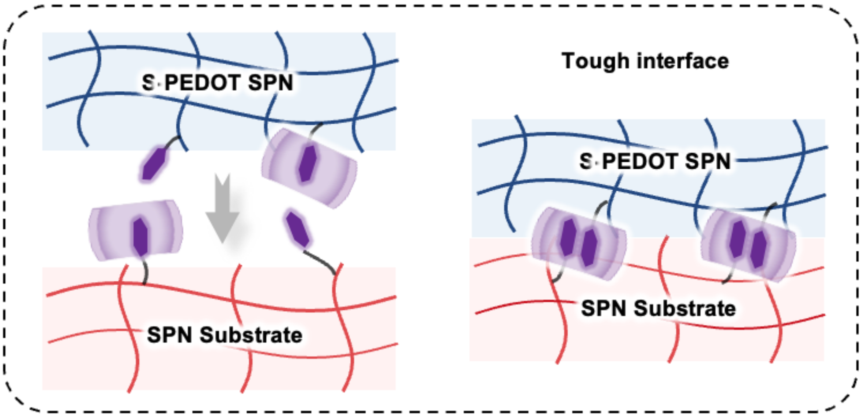

Figure S12: Interfacial adhesion of S-PEDOT SPN to the underlying substrate layer. Interfacial crosslinking at the interface between the S-PEDOT SPN and SPN substrate, on account of the high-binding affinity supramolecular complexes used in both materials, leads to strong adhesion without any delamination during use.

S.11 Comparison to previous reports of conducting polymer hydrogels

Table S1: Table comparing conductivity, Young's modulus, stretchability and toughness of the S-PEDOT SPN compared to previous reports of conductive polymeric hydrogels.

Polymer	Young's modulus kPa	Conductivity S cm^{-1}	Stretchability %	Toughness kPa	Reference
PPy	300	0.012	80	10.5	3
	2.2	0.4	9	0.005	4
	13	0.12	1250	375	5
	146	0.026	628	1100	6
	140	0.8	375	188	7
PANI	47	0.04	133	6.5	8**
	24	0.0062	70	3.8	9
	100	0.05	50	12.5	10
	119.8	0.006	125	92.9	11
	3	0.13	700	73.5	12
PEDOT:PSS	110	0.022	817	1600	13
	15	0.1	50	2.4	14*
	2100	40	30	180	15
	2970	1	75	800	16
	374	0.23	121	151	17*
	4	0.52	534	160	1
	460	10	140	440	18*
	60	247	400	977	19*
	920	11	400	2000	20*
PEDOT:PSS/PANI	14.1	3	355	63	21
**S-PEDOT**	**10.5**	**5.8**	**1070**	**630**	**this work**

* = highest reported conductivity sample was chosen as benchmark for properties comparison.
** = Conductivity value in water.

References

[1] S. J. O'Neill, Z. Huang, M. H. Ahmed, A. J. Boys, S. Velasco-Bosom, J. Li, R. M. Owens, J. A. McCune, G. G. Malliaras, O. A. Scherman, *Adv. Mater.* **2023**, *35*, 1 2207634.

[2] H. Yano, K. Kudo, K. Marumo, H. Okuzaki, *Sci. Adv.* **2019**, *5*, 4 eaav9492.

[3] L. Wang, J. Jiang, W. Hua, A. Darabi, X. Song, C. Song, W. Zhong, M. M. Xing, X. Qiu, *Adv. Funct. Mater.* **2016**, *26* 4293.

[4] E. Chalmers, H. Lee, C. Zhu, X. Liu, *Chem. Mater.* **2020**, *32* 234.

[5] L. Han, L. Yan, M. Wang, K. Wang, L. Fang, J. Zhou, J. Fang, F. Ren, X. Lu, *Chem. Mater.* **2018**, *30* 5561.

[6] L. Zhao, X. Li, Y. Li, X. Wang, W. Yang, J. Ren, *Biomacromolecules* **2021**, *22* 1273.

[7] X. Yang, L. Cao, J. Wang, L. Chen, *ACS Sustainable Chemistry & Engineering* **2020**, *8*, 29 10726.

[8] J. Stejskal, P. Bober, M. Trchova, A. Kovalcik, J. Hodan, J. Hromadkova, J. Prokes, *Macromolecules* **2017**, *50* 972–978.

[9] C. Zhang, M.-H. Hsieh, S.-Y. Wu, S.-H. Li, J. Wu, S.-M. Liu, H.-J. Wei, R. D. Weisel, H.-W. Sung, R.-K. Li, *Biomaterials* **2018**, *231* 11967.

[10] Z. Wang, H. Zhou, J. Lai, B. Yan, H. Liu, X. Jin, A. Ma, G. Zhang, W. Zhao, W. Chen, *J. Mater. Chem. C* **2018**, *6* 9200–9207.

[11] Y. Zhao, B. Zhang, B. Yao, Y. Qiu, Z. Peng, Y. Zhang, Y. Alsaid, I. Frenkel, K. Youssef, Q. Pei, et al., *Matter* **2020**, *3* 1196–1210.

[12] J. Chen, Q. Peng, T. Thundat, H. S. Zeng, *Chem. Mater.* **2019**, *31* 4553.

[13] Q. Wu, J. Wei, B. Xu, X. Liu, H. Wang, W. Wang, Q. Wang, W. Liu, *Sci. Reports* **2017**, *2017*, 71 7.

[14] S. Zhang, Y. Chen, H. Liu, Z. Wang, H. Ling, C. Wang, J. Ni, B. Celebi-Saltik, X. Wang, X. Meng, et al., *Adv. Mater.* **2020**, *32*, 19047 52.

[15] B. Lu, H. Yuk, S. Lin, N. Jian, K. Qu, J. Xu, X. Zhao, *Nat. Commun.* **2019**, *10* 1.

[16] R. Kishi, K. Kubota, T. Miura, T. Yamaguchi, H. Okuzaki, Y. Osada, *J. Mater. Chem. C* **2014**, *2* 736.

[17] V. R. Feig, H. Tran, M. Lee, Z. Bao, *Nat. Commun.* **2018**, *9* 1.

[18] G. Li, K. Huang, J. Deng, M. Guo, M. Cai, Y. Zhang, C. F. Guo, *Adv. Mater.* **2022**, *34*, 15 2200261.

[19] J. Chong, C. Sung, K. S. Nam, T. Kang, H. Kim, H. Lee, H. Park, S. Park, J. Kang, *Nat. Commun.* **2023**, *14*, 1 2206.

[20] T. Zhou, H. Yuk, F. Hu, J. Wu, F. Tian, H. Roh, Z. Shen, G. Gu, J. Xu, B. Lu, et al., *Nat. Mater.* **2023**, 1–8.

[21] Y. Shin, H. S. Lee, Y. J. Hong, S.-H. Sunwoo, O. K. Park, S. H. Choi, D.-H. Kim, S. Lee, *Sci. Adv.* **2024**, *10*, 12 7724.
